# Increased Risk of Polycystic Ovary Syndrome and It’s Comorbidities in Women with Autoimmune Thyroid Disease

**DOI:** 10.3390/ijerph17072422

**Published:** 2020-04-02

**Authors:** Chun-Wei Ho, Hsin-Hung Chen, Ming-Chia Hsieh, Ching-Chu Chen, Sheng-Pang Hsu, Hei-Tung Yip, Chia-Hung Kao

**Affiliations:** 1 Intelligent Diabetes Metabolism and Exercise Center, China Medical University Hospital, Taichung 40447, Taiwan; d34914@mail.cmuh.org.tw (C.-W.H.); d34913@mail.cmuh.org.tw (H.-H.C.); d94750@mail.cmuh.org.tw (M.-C.H.); 2 School of Medicine, Institute of Medicine and Public Health, Chung Shan Medical University, Taichung 40201, Taiwan; 3 Division of Clinical Nutrition, China Medical University Hospital, Taichung 40447, Taiwan; 4 Graduate Institute of Integrative Medicine, China Medical University, Taichung 40447, Taiwan; 5 Division of Endocrinology and Metabolism, Department of Internal Medicine, China Medical University Hospital, Taichung 40447, Taiwan; d1918@mail.cmuh.org.tw (C.-C.C.); ilms.always03@gmail.com (S.-P.H.); 6 School of Chinese Medicine, China Medical University, Taichung 40447, Taiwan; 7 Management Office for Health Data, China Medical University Hospital, Taichung 40447, Taiwan; fionyip0i0@gmail.com; 8 College of Medicine, China Medical University, Taichung 40447, Taiwan; 9 Graduate Institute of Biomedical Sciences and School of Medicine, College of Medicine, China Medical University, Taichung 40447, Taiwan; 10 Department of Nuclear Medicine and PET Center, China Medical University Hospital, Taichung 40447, Taiwan; 11 Department of Bioinformatics and Medical Engineering, Asia University, Taichung 40447, Taiwan; 12 Center of Augmented Intelligence in Healthcare, China Medical University Hospital, Taichung 40447, Taiwan

**Keywords:** polycystic ovary syndrome (PCOS), autoimmune thyroid disease (AITD), hashimoto thyroiditis (HT), grave disease (GD)

## Abstract

Objective: To investigate the prevalence of polycystic ovary syndrome (PCOS) and its comorbidities in patients with autoimmune thyroid disease (AITD). Population: In this cohort study, patients newly diagnosed as having Hashimoto thyroiditis (HT) or Grave disease (GD) were recruited into the AITD group. Method: The logistic regression model was used to investigate the association between exposure, endpoint, later diseases and treatment. Main Outcome Measures: We assessed the cumulative incidence using the Kaplan–Meier method and verified the difference by the log-rank test. Results: The AITD group included 3599 GD patients and 1332 HT patients. PCOS risk in patients with AITD was higher than that in the control group (adjusted hazard ratio = 1.39; 95% confidence interval = 1.07–1.71). In patients with both AITD and PCOS, the odds ratios of diabetes, hyperlipidemia and coronary artery disease were 2.48, 2.05 and 2.63, respectively. Conclusions: The risks of PCOS and its comorbidities such as diabetes, dyslipidemia and cardiac artery disease are high in patients with AITD in Taiwan.

## 1. Introduction

Autoimmune thyroid disease (AITD) is one of the most prevalent autoimmune diseases in the general population [1,2]. AITDs, including Hashimoto thyroiditis (HT) and Grave disease (GD), affect 10–20% of all women. AITD may occur due to the generation of thyroid autoantibodies with an abnormal thyroid hormone production as well as T- and B-cell infiltration into the thyroid gland [3]. GD may be caused by generating a B-cell immune response to the thyroid-stimulating hormone (TSH) receptor antibody, causing thyroid follicular cell hyperplasia and hyperthyroidism. HT may be triggered by a T-cell-mediated immune response, resulting in thyroid destruction and causing overt hypothyroidism [4]. Both environmental and genetic factors may be involved in AITD etiology. HT and GD share similar immune-mediated mechanisms [5]. Polycystic ovary syndrome (PCOS) is the most common endocrine disorder in women of reproductive age [6]. A review article reported that PCOS can induce a disruption of the hypothalamic-pituitary-gonadal axis, dysregulation of ovarian steroidogenesis, low-grade chronic inflammation and hyperinsulinemia, possibly resulting from genetic and environmental factors. On the basis of these mechanistic theories, PCOS could be associated with diabetes, obesity, dyslipidemia, hypertension (HTN) and other cardiovascular diseases. Even after geographical stratification, high AITD risk persists in women with PCOS; the proportion is particularly higher in Asian women than in European and American women [7]. Numerous studies have reported the effect of thyroid disease on PCOS for women of reproductive age, but to our knowledge, only a few studies have explored PCOS and its comorbidities in the AITD population in Asia. Here, we evaluated the risk of PCOS and its comorbidities in patients with AITD.

## 2. Methods

### 2.1. Data Source

The data source was the Taiwan National Health Insurance (NHI) Research Database (NHIRD), established in 1995; it contains the medical records of almost 99% of Taiwan’s residents. We analyzed the Longitudinal Health Insurance Database (LHID), a subset of the NHIRD, to investigate the association between AITD and PCOS. The disease code was defined according to the International Classification of Diseases, Ninth Revision, Clinical Modification (ICD-9-CM). The patient identification data in the LHID are encrypted to protect patient privacy.

### 2.2. Patient Selection

In this cohort study, patients newly diagnosed as having HT (ICD-9-CM 245.21) or GD (ICD-9-CM 242.0) were recruited in the AITD group. The index date was defined as the date of HT and GD diagnosis. People without a history of HT or GD were defined as the non-AITD group. The non-AITD individuals were assigned a random index date between 2000 and 2013. Patients who received a diagnosis of PCOS before the index date or were aged <18 years were excluded. All patients were followed from the index date to the date of primary outcome occurrence, withdrawal from the NHI program or the end of the study.

### 2.3. Main Outcome, Comorbidities and Medication

The event of interest for this study was the PCOS (ICD-9-CM 256.4). PCOS patients were defined by having at least two out-patient and one in-patient visits. We further confirmed patients with the outcome of PCOS by verifying the execution of the related blood test and ultrasound scan. The following comorbidities were considered as potential confounders: diabetes (ICD-9-CM 250), HTN (ICD-9-CM 401–405), hyperlipidemia (ICD-9-CM 272), stroke (ICD-9-CM 430-438), coronary artery disease (CAD; ICD-9-CM 410-414) and heart failure (HF; ICD-9-CM 428). We defined patients who had ever taken thyroxine or anti-thyroid drugs(ATD) (i.e., methimazole, carbimazole and propylthiouracil) as drug treatment receivers.

### 2.4. Statistical Analysis

To examine the baseline characteristic differences between the AITD and non-AITD groups, we applied the chi-square test for categorical variables and the Student *t* test for continuous variables. The PCOS incidence rate for different variables was calculated and the hazard ratio for the PCOS was estimated using the Cox proportional hazard regression model.

We adjusted the hazard ratios by including age and all comorbidities in the regression model. The logistic regression model was used to investigate the association between exposure, endpoint and later diseases. The effects of medical treatments on later diseases were also analyzed. We assessed the cumulative incidence rate of PCOS in the AITD and non-AITD groups by using the Kaplan–Meier method and verified the difference using the log-rank test.

### 2.5. Ethics Approval and Consent to Participate

The NHIRD encrypts patient personal information to protect privacy and provides researchers with anonymous identification numbers associated with relevant claims information, including sex, date of birth, medical services received and prescriptions. Therefore, patient consent is not required to access the NHIRD. This study was approved to fulfill the condition for exemption by the Institutional Review Board (IRB) of China Medical University (CMUH104-REC2-115-CR4).

## 3. Results

In our cohort study, 9655 patients satisfied the inclusion criteria, comprising 6731 patients with AITD; moreover, 26,924 controls were included. In the AITD group, 5399 and 1332 patients had GD and HT, respectively. The mean follow-up duration was 7.40 (±3.81) and 7.30 (±3.80) years in the AITD and non-AITD groups, respectively. Table 1 displays the baseline characteristics of the AITD and non-AITD groups. In the AITD group, the mean age was 43.7 (±16.5) years—higher than that in the non-AITD group (42.3 (±14.5)years). The distributions of HTN, stroke, CAD and HF between the AITD patients and non-AITD patients displayed no significant differences. Diabetes and hyperlipidemia prevalence was higher in the AITD group than in the non-AITD group (*p* < 0.001).

**Figure 1:**
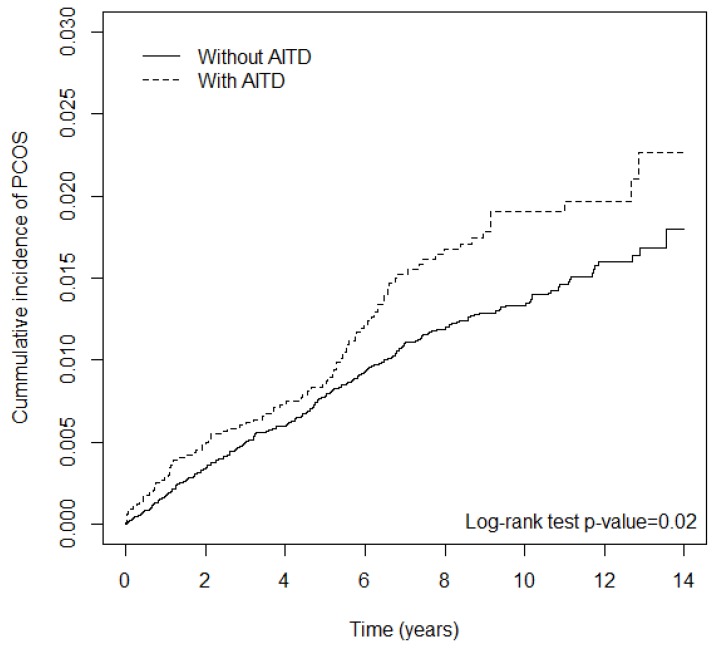
The cumulative incidence of polycystic ovary syndrome(PCOS) in the patients with and without autoimmune thyroid disease (AITD).

Figure 1 demonstrates that the cumulative incidence of PCOS in the AITD patients was higher than that in non-AITD patients (*p* = 0.02). The PCOS risk in patients with AITD was higher than that in the comparison group (adjusted hazard ratio = 1.39; 95% confidence interval = 1.07–1.71). Older patients had a lower PCOS risk. The hazard ratio of PCOS for patients with any comorbidity, relative to patients without any comorbidities, was 1.59 (95% confidence interval = 1.10–2.30) (Table 2).

The relationships between the comorbidities after the index date and AITD or PCOS are presented in Table 3. We considered the patients without AITD or PCOS as the reference group. Patients with AITD demonstrated an increased risk of diabetes, hypertension, hyperlipidemia, stroke, CAD and HF. In patients with both AITD and PCOS, the odds ratios of diabetes, hyperlipidemia and CAD were 2.48, 2.05 and 2.63, respectively.

Table 4 displays the association between the medication and the comorbidities after the index date. When considering individuals without AITD, PCOS and medication as a reference, AITD and PCOS patients who used the medication did not significantly increase the incidence of later diseases.

## 4. Discussion

A Danish study reported that the hazard ratio for thyroid disease development was 2.5 times higher in patients with PCOS [8]. Another report indicated a three-fold higher prevalence of AITD in patients with PCOS [9]. In an Italian study, AITD prevalence in patients with PCOS was significantly higher (27% vs. 8%) but no other autoimmune disease was associated with PCOS [10]. AITD was observed at a higher rate in the patients with PCOS compared with the control groups (26.03% vs. 9.72%). A significant odds ratio of 3.27 was demonstrated between PCOS and the chance of AITD. After geographical stratification, the high risk of AITD in women with PCOS significantly persisted for Asians compared with Europeans and South Americans (ORs = 4.56, 3.27 and 1.86 respectively) [7]. We determined that the PCOS risk in patients with AITD was 1.39-fold higher than that in the control group but 4.56-fold lower than the AITD risk in patients with PCOS in Asia [7]. We also did the analysis of HT and GD for AITD risk. The result showed that the PCOS risk in patients with HT was 1.63-fold higher but in patients with GD was 1.24-fold higher. Our findings support the established common mechanism between PCOS and AITD. Androgen excess was noted in PCOS, which could induce the reduction of most immune system elements, for instance, enhancing T suppressor cell activity or promoting TH1 responses [11]. In thyroid disease, abnormal interactions due to environmental or hormonal factors were observed between thyrocytes and T cells. In HT, TH1-mediated autoimmunity reportedly causes the lysis of thyrocytes and hypothyroidism. In GD, stimulatory TH2 responses against the TSH receptors reportedly cause hyperthyroidism [12]. According to large epidemiological reports, AITD is the most frequent cause of hypothyroidism or subclinical hypothyroidism in the adult population [13,14]. In women with PCOS, subclinical hypothyroidism was reported with an estimated prevalence of approximately 10% to 25%, which could progress to overt hypothyroidism [13,15]. A recent meta-analysis reported that the coexistence of subclinical hypothyroidism and PCOS caused alterations in serum lipids and insulin resistance [16]. In our study, patients with GD were at higher risk than those with HT, which indicates that the relationship between AITD and PCOS is not only due to hypothyroidism but also other underlying factors. A nationwide Danish study for morbidity and medicine prescriptions for people with PCOS revealed a higher prevalence of thyroid disease, diabetes, dyslipidemia, hypertension and a two-fold increased risk of stroke and thrombosis, but did not display an increased risk of other cardiovascular diseases [17]. In our study, patients with both AITD and PCOS had odds ratios of diabetes, hyperlipidemia and CAD of 2.48, 2.05 and 2.63, respectively, but did not display an increased stroke risk. The diabetes risk was estimated to be 5–10 times increased in patients with PCOS, but these findings are controversial [18,19,20,21]. In our analysis, the diabetes risk was lower than in the aforementioned Danish study. In our analysis, we found that all of our comorbidities were elevated in patients with AITD alone instead of patients with PCOS alone. According to other PCOS studies, complications or comorbidities such as cardio-metabolic risk, obesity or insulin resistance in patients with PCOS are common [22,23]. Further research concerning the different race issue only or other basic mechanisms for AITD with PCOS should be done in the future. Importantly, we should pay more attention to these women combining AITD and PCOS for the screen of diabetes, dyslipidemia and CAD.

### Limitations

First, laboratory data on the follicle-stimulating hormone, luteinizing hormone, insulin levels and thyroid function were unavailable in the NHIRD. Second, image data, such as thyroid and ovary echograms, were also unavailable in the NHIRD. Third, nutritional status or records, such as weight, body mass index, iodine levels in diet, exercise level, smoking and alcohol consumption, were also unavailable. Nevertheless, because the NHI program provides an efficient service to patients with AITD and PCOS with endocrinologists and gynecologists, the accuracy of the diagnosis of AITD or PCOS is high.

## 5. Conclusions

Our study analyzed only a few factors and comorbidities of PCOS in patients with AITD. The results indicate an increased risk of PCOS and comorbidities, such as diabetes, dyslipidemia and CAD in patients with AITD in Taiwan.

## Figures and Tables

**Table 1:** The baseline characteristics of the autoimmune thyroid disease (AITD) patients and controls.

Variables	AITD				
	No		Yes		
	(*N* = 26,924)		(*N* = 6731)		
Variables	*n*	%	*n*	%	*p*-Value
Age					1.00
<30	6132	22.8%	1533	22.8%	
30–39	6836	25.4%	1709	25.4%	
≥40	13,956	51.8%	3489	51.8%	
Mean (SD) ^+^	42.3	(14.5)	43.7	(16.5)	<0.001
Comorbidities					
Diabetes (DM)	1602	6.0%	493	7.3%	<0.001
Hypertension (HTN)	2373	8.8%	623	9.3%	0.26
Hyperlipidemia	2706	10.1%	950	14.1%	<0.001
Stroke	1206	4.5%	293	4.4%	0.68
Coronary artery disease (CAD)	1219	4.5%	336	5.0%	0.11
Heart failure	667	2.5%	173	2.6%	0.69

^+^: student *t*-test.

**Table 2:** The incidence rate and the hazard ratios of polycystic ovary syndrome (PCOS).

Variables	PCOS						
	Events	py	Rate	Crude HR	(95% CI)	Adjusted HR	(95% CI)
AITD							
No	280	196,598	1.42	1.00	(Reference)	1.00	(Reference)
Yes	94	49,790	1.89	1.33	(1.05, 1.68) **	1.39	(1.07, 1.71) *
GD							
No	280	196,598	1.42	1.00	(Reference)	1.00	(Reference)
Yes	75	40,873	1.83	1.29	(1.00, 1.67) *	1.24	(0.96, 1.60)
HT							
No	280	196,598	1.42	1.00	(Reference)	1.00	(Reference)
Yes	19	8917	2.13	1.48	(0.93, 2.36)	1.63	(1.02, 2.60) *
Age							
<30	250	60,044	4.16	1.00	(Reference)		
30–39	114	65,368	1.74	0.42	(0.34, 0.52) ***		
≥40	10	120,975	0.08	0.02	(0.01, 0.04) ***		
Comorbidities							
No	338	179,975	1.88	1.00	(Reference)	1.00	(Reference)
Yes	36	66,413	0.54	0.28	(0.20, 0.40) ***	1.59	(1.10, 2.30) *

py: person-years; rate: incidence rate (per 1000 py); adjusted HR: adjusted with age and comorbidities of DM, HTN, hyperlipidemia, stroke, CAD and heart failure; HT: Hashimoto thyroiditis; GD: Grave disease; * *p* < 0.05, ** *p* < 0.01, *** *p* < 0.001.

**Table 3:** The distribution of later diseases in the patients with and without AITD and PCOS.

Later Diseases		Number of Patients	Number of Events	Adjusted OR	(95% CI)
DM					
AITD	PCOS				
No	No	26,623	1588	1.00	(Reference)
Yes	No	6637	486	1.36	(1.22, 1.52) ***
No	Yes	280	14	1.71	(0.99, 2.95)
Yes	Yes	94	7	2.48	(1.14, 5.38) *
HTN					
AITD	PCOS				
No	No	26,623	2355	1.00	(Reference)
Yes	No	6637	616	1.11	(1.01, 1.22) *
No	Yes	280	18	1.15	(0.71, 1.86)
Yes	Yes	94	7	1.30	(0.60, 2.81)
Hyperlipidemia					
AITD	PCOS				
No	No	26,623	2683	1.00	(Reference)
Yes	No	6637	938	1.56	(1.44, 1.69) ***
No	Yes	280	23	1.30	(0.85, 2.01)
Yes	Yes	94	12	2.05	(1.11, 3.77) *
Stroke					
AITD	PCOS				
No	No	26,623	1204	1.00	(Reference)
Yes	No	6637	292	1.16	(1.02, 1.33) *
No	Yes	280	2	0.58	(0.14, 2.36)
Yes	Yes	94	1	0.79	(0.11, 5.71)
CAD					
AITD	PCOS				
No	No	26,623	1218	1.00	(Reference)
Yes	No	6637	331	1.22	(1.08, 1.39) **
No	Yes	280	1	0.18	(0.03, 1.27)
Yes	Yes	94	5	2.63	(1.06, 6.51) *
Heart failure					
AITD	PCOS				
No	No	26,623	667	1.00	(Reference)
Yes	No	6637	173	1.39	(1.16, 1.66) ***
No	Yes	280	0	-	-
Yes	Yes	94	0	-	-

Adjusted odd ratio(OR): adjusted with age; * *p* < 0.05, ** *p* < 0.01, *** *p* < 0.001.

**Table 4:** The distribution of later diseases in the AITD and PCOS patients with and without drug usage.

Later Diseases		Number of Patients	Number of Events	Adjusted OR	(95% CI)
DM					
AITD + PCOS	Drugs				
No	No	26,644	1588	1.00	(Reference)
Yes	Yes	29	2	2.31	(0.55, 9.76)
HTN					
AITD + PCOS	Drugs				
No	No	26,644	2989	1.00	(Reference)
Yes	Yes	29	5	1.20	(0.28, 5.06)
Hyperlipidemia					
AITD + PCOS	Drugs				
No	No	26,644	2683	1.00	(Reference)
Yes	Yes	29	2	1.05	(0.25, 4.43)
Stroke					
AITD + PCOS	Drugs				
No	No	26,644	1204	1.00	(Reference)
Yes	Yes	29	0	-	-
CAD					
AITD+ PCOS	Drugs				
No	No	26,644	1218	1.00	(Reference)
Yes	Yes	29	1	1.72	(0.23, 12.7)
Heart failure					
AITD + PCOS	Drugs				
No	No	26,644	667	1.00	(Reference)
Yes	Yes	29	0	-	-

Adjusted OR: adjusted with age
